# Interactions between an anti-sigma protein and two sigma factors that regulate the pyoverdine signaling pathway in *Pseudomonas aeruginosa*

**DOI:** 10.1186/s12866-014-0287-2

**Published:** 2014-11-30

**Authors:** Rebecca J Edgar, Xin Xu, Matt Shirley, Anna F Konings, Lois W Martin, David F Ackerley, Iain L Lamont

**Affiliations:** Department of Biochemistry, University of Otago, PO Box 56 Dunedin, New Zealand; School of Biological Sciences, Victoria University of Wellington, PO Box 600, Wellington, New Zealand

**Keywords:** Siderophore, Gene expression, ECF sigma factor, Anti-sigma factor, Pyoverdine, *Pseudomonas*, Cell surface signaling, TonB-dependent signaling, Tandem affinity purification

## Abstract

**Background:**

Synthesis and uptake of pyoverdine, the primary siderophore of the opportunistic pathogen *Pseudomonas aeruginosa*, is dependent on two extra-cytoplasmic function (ECF) sigma factors, FpvI and PvdS. FpvI and PvdS are required for expression of the ferri-pyoverdine receptor gene *fpvA* and of pyoverdine synthesis genes respectively. In the absence of pyoverdine the anti-sigma factor FpvR that spans the cytoplasmic membrane inhibits the activities of both FpvI and PvdS, despite the two sigma factors having low sequence identity.

**Results:**

To investigate the interactions of FpvR with FpvI and PvdS, we first used a tandem affinity purification system to demonstrate binding of PvdS by the cytoplasmic region of FpvR in *P. aeruginosa* at physiological levels. The cytoplasmic region of FpvR bound to and inhibited both FpvI and PvdS when the proteins were co-expressed in *Escherichia coli*. Each sigma factor was then subjected to error prone PCR and site-directed mutagenesis to identify mutations that increased sigma factor activity in the presence of FpvR. In FpvI, the amino acid changes clustered around conserved region four of the protein and are likely to disrupt interactions with FpvR. Deletion of five amino acids from the C-terminal end of FpvI also disrupted interactions with FpvR. Mutations in PvdS were present in conserved regions two and four. Most of these mutations as well as deletion of thirteen amino acids from the C-terminal end of PvdS increased sigma factor activity independent of whether FpvR was present, suggesting that they increase either the stability of PvdS or its affinity for core RNA polymerase.

**Conclusions:**

These data show that FpvR binds to PvdS in both *P. aeruginosa* and *E. coli*, inhibiting its activity. FpvR also binds to and inhibits FpvI and binding of FpvI is likely to involve conserved region four of the sigma factor protein.

**Electronic supplementary material:**

The online version of this article (doi:10.1186/s12866-014-0287-2) contains supplementary material, which is available to authorized users.

## Background

*Pseudomonas aeruginosa* is a widespread opportunistic pathogen recognized for its role in morbidity and mortality in cystic fibrosis and burns patients [1]. Like other bacteria *P. aeruginosa* has a requirement to take up iron, which is an essential co-factor in a number of proteins. *P. aeruginosa* achieves this via active uptake of iron-chelating siderophores, with pyoverdine being the primary siderophore secreted by this bacterium [2]. Once pyoverdine has bound iron, the cell-surface receptor FpvA binds and transports ferri-pyoverdine into the cell (reviewed in [3]). Expression of pyoverdine synthesis genes and the *fpvA* gene is directed by the alternative sigma factors PvdS and FpvI respectively [4-7], and PvdS is also required for maximal expression of two secreted virulence factors, exotoxin A and PrpL protease [8,9]. In the absence of pyoverdine the activities of PvdS and FpvI are inhibited by an anti-sigma factor, FpvR, which spans the cytoplasmic membrane [6,10,11]. In a positive feedback loop, interaction of ferri-pyoverdine with FpvA results in proteolytic degradation of FpvR, a process that requires the energy-transducing protein TonB as well as interaction between periplasmic domains of the Fpv proteins [12-15]. The sigma factors are then free to recruit core RNA polymerase, facilitating promoter recognition with consequent up-regulation of the pyoverdine synthesis genes and the genes encoding FpvA, exotoxin A and PrpL protease. Signal transduction systems of this sort (cell-surface signaling) are widespread in Gram negative bacteria and control the expression of a large number of genes encoding ferri-siderophore receptor proteins, in a wide range of species [11,16,17]. However the ferri-pyoverdine system is the only cell-surface signaling pathway known in which a single anti-sigma factor (FpvR) inhibits two different sigma factors (PvdS and FpvI). In some cell-surface signaling pathways, such as the Fec (ferric citrate) pathway in *Escherichia coli* [18] and the Fox (desferrioxamine) and Fiu (ferrichrome) pathways in *P. aeruginosa* [19] the anti-sigma factors are also required for sigma factor function and so are considered to be sigma factor regulators. However, there is no evidence that FpvR is required for activity of PvdS or FpvI.

FpvI and PvdS belong to the class IV or extra-cytoplasmic function (ECF) sigma factors, alternative sigma factors that control a wide range of functions in bacteria and are the largest and most diverse group of sigma factors known (reviewed in [20,21]). Class IV sigma factors are relatively small and share only two of the four conserved functional regions present in other sigma factors, region two and region four, these being connected by a flexible non-conserved linker. Region two plays a specific role in −10 promoter recognition and in DNA melting, and region four recognizes the −35 promoter region [20]. In a previous study, alanine scanning mutagenesis was used to elicit more detail on the functions of these regions in PvdS [22]. Mutations in region 2.1 and 2.2 reduced binding to core RNA polymerase and mutations in regions 2.3, 2.4 and 4.2 impaired DNA binding without affecting binding to core RNA polymerase. It is very likely that the corresponding regions in FpvI have equivalent functions.

Binding of sigma factors by anti-sigma factor proteins provides an effective mechanism of post-translational control of protein activity in cell-surface signaling and many other bacterial systems. However, there are very few cases in which sigma/anti-sigma factor interactions have been characterized at the molecular level and none of these closely parallel the FpvR/FpvI/PvdS system. One well-studied example is the stress response sigma factor σ^E^ in complex with the N-terminal (residues 1–66) region of its anti-sigma factor RseA from *E. coli* [23]. The region RseA_N1–66_ slots between regions two and four of σ^E^, making extensive interactions that sterically prevent σ^E^ from recruiting core RNA polymerase. The cytoplasmic domain of the anti-sigma factor ChrR from *Rhodobacter sphaeroides* has a similar structure to RseA_N1–66_ [24]. This observation in combination with bioinformatic analysis has led to the proposal that there is a common structure for the cytoplasmic domain of class IV anti-sigma factors [24], despite low sequence identity. However, there is considerable variability amongst ECF sigma factors for the regions bound by the cognate anti-sigma factor, which can be both region two and four [23-25], region two alone [26] or region four alone [27].

Although both are inhibited by FpvR, FpvI and PvdS have low sequence identity with each other (34.7%) [6]. Previously, bacterial 2-hybrid analysis demonstrated an interaction between FpvI and FpvR, and PvdS and FpvR, when each pair was expressed in *E. coli* [28]. It was also shown that the cytoplasmic N-terminal 67 amino acids of FpvR comprised the minimum region required for interaction with PvdS and FpvI [28]. Mutations were identified in *fpvI* that interfered with the interaction of FpvR and FpvI although the effects of most of these mutations were attributed to reduced amounts of FpvI protein, with only one mutation specifically affecting binding of FpvI by FpvR. The effect of mutations in PvdS on interactions with FpvR was not investigated.

The overall aim of the research described here was to investigate the interactions of FpvR with PvdS and FpvI *in vivo*, and to identify amino acid residues in each of the sigma factors that contribute to those interactions.

## Results

### Co-purification of FpvR and PvdS from *P. aeruginosa*

**Figure 1:**
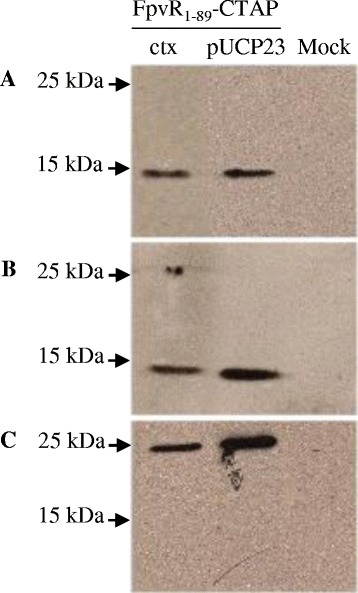
**Co-purification of PvdS with FpvR**_**1–89**_ **–TAP from** ***P. aeruginosa*** **.** Soluble protein was prepared from *P. aeruginosa* PAO1 *fpvR* expressing plasmid-borne (pUCP23) or chromosomally-integrated (ctx) FpvR_1–89_ fused to a C-terminal TAP tag. Protein was purified using the TAP protocol and the purified protein analyzed by Western blotting for FpvR_1–89_-CBP or PvdS. **(A)** anti-CBP; **(B)** anti-FpvR; **(C)** anti-PvdS. A mock purification was carried out with *P. aeruginosa* PAO1 *fpvR* carrying pUCP23 without the *fpvR*_*1–89*_-TAP fusion as a negative control for the TAP tag purification procedure. The positions of molecular weight markers are shown.

We validated binding of PvdS by FpvR in *P. aeruginosa* by purifying the cytoplasmic portion of FpvR and determining whether PvdS was co-purified. *P. aeruginosa* (PAO1) was engineered to express the cytoplasmic portion and predicted sigma factor inhibitory region of FpvR (residues 1–89) [6,28] fused to a C-terminal tandem affinity purification (TAP) tag [29]. The FpvR_1–89_ -TAP fusion, expressed from the *fpvR* promoter, was either integrated into the bacterial chromosome using mini-CTX or was expressed from plasmid pUCP23. Chromosomal integration was used to demonstrate that FpvR_1–89_ and PvdS interact when expressed in physiological amounts. Higher plasmid-based expression was expected to titrate out any regulatory factors that may have limited FpvR_1–89_ expression, ensuring sufficient FpvR_1–89_ was present for visualisation and co-purification with PvdS. As expected, expression of FpvR from the chromosomally-integrated construct was repressed by the presence of iron in the King’s B medium (Additional file 1: Figure S1). FpvR_1–89_ fused to calmodulin binding protein (CBP) was purified using the TAP protocol. The purification resulted in a 15 kDa protein, the predicted size for FpvR_1–89_–CBP, which could be detected using antibodies against either CBP or FpvR_1–89_ (Figure 1). Fractions that contained purified FpvR_1–89_–CBP also contained co-purified PvdS. PvdS was not present in fractions obtained using the purification protocol with bacteria that did not contain the FpvR_1–89_–TAP construct, confirming that purification of PvdS was dependent on the presence of FpvR_1–89_–TAP. FpvI was not detected following FpvR_1–89_–TAP purification using a polyclonal FpvI antibody and a suitable monoclonal antibody was not available.

### The cytoplasmic region of FpvR inhibits sigma factor activity in *E. coli*

**Figure 2:**
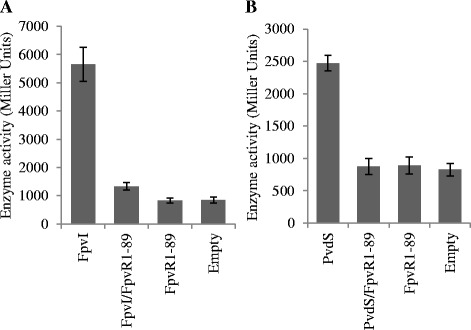
**The activity of PvdS and FpvI in the presence and absence of FpvR**_**1–89**_ **.** β-galactosidase assays were carried out with *E. coli* MC1061 (DE3) containing either **(A)** pMP190::P_*fpvA*__*lacZ* or **(B)** pMP190::P_*pvdE*__*lacZ*, along with pETDuet expressing FpvR_1–89_ and either **(A)** FpvI or **(B)** PvdS. An empty pETDuet control is also shown to control for background expression of *lacZ* from pMP190::P_*fpvA*__*lacZ* or pMP190::P_*pvdE*__*lacZ*. Averages were obtained from three biological replicates. Error bars are ±1 SD.

To investigate the inhibition of FpvI and PvdS by FpvR_1–89_, a system was established for detecting the activity of FpvI or PvdS when co-expressed with FpvR_1–89_ in *E. coli*. The pETDuet vector allows a 1:1 molar ratio of expression from two multiple cloning sites (MCS). DNA encoding *fpvR*_*1–89*_ was inserted into MCS2 and either *fpvI* or *pvdS* was inserted into MCS1, giving plasmids pETDuet*fpvI*_*fpvR*_*1–89*_ and pETDuet*pvdS*_*fpvR*_*1–89*_ respectively (Table 1). Reporter plasmids (Table 1) carrying *fpvA* or *pvdE* promoters fused upstream of *lacZ* were used to detect FpvI or PvdS activity respectively [6,30]. In the absence of FpvR_1–89_, FpvI induced *fpvA* promoter activity and PvdS induced *pvdE* promoter activity (Figure 2). The activity of FpvI and PvdS was strongly repressed in the presence of FpvR_1–89_. These results demonstrated the inhibitory function of the cytoplasmic region of FpvR on both FpvI and PvdS activity in *E. coli*.

**Table 1:** **Bacterial strains and plasmids used in this study**

**Strains**	**Genotype/phenotype**	**Reference**
*P. aeruginosa*		
PAO1	Wild-type	[31]
PAO1 *fpvR*	PAO1 with *fpvR* (PA2388) deletion	[10]
PAO1 *fpvR*_1–89_-TAP	PAO1 containing mini CTX2:: *fpvR*_*1–89*_-TAP	This study
PAO1*fpvR*; pUCP23::*fpvR*_1–89_-TAP	PAO1*fpvR* containing pUCP23::*fpvR*_1–89_-TAP; Gm^R^, Cb^R^	This study
*E. coli*		
S17-1	*hsd* ^*R*^ *hsdM* ^+^ *recA thi pro* [integrated *RP*4-2-*Tc*::*Mu*, *Km*::*Tn7*]; *Sm* ^R^ *Tp* ^R^	[32]
JM83	F- *ara* Δ (*lac-proAB*) *rpsL* [Φ*80, lacZ*Δ*M15*] *thi*	[33]
MC1061	Δ*lacX*74 Hsr^−^ Hsm^+^ *rpsL*	[34]
MC1061 (DE3)	MC1061 lysogenized with λDE3 [*lacI lac*UV5-T7 gene 1]	This study
*Plasmids*		
pUCP23	*lacI* ^*q*^ *lacZ*(α-fragment) *aacC1* ColE1 ori; RO1600 ori; Cb/Amp^R^, Gm^R^	[35]
pUCP23::*fpvR*_1-89_TAP	519 bp PCR fragment containing the *fpvR* promoter and 5′end of *fpvR*, joined to a C-terminal TAP-tag and cloned into pUCP23	This study
Mini-CTX2	Self-proficient integration vector; Tc^R^	[36]
Mini-CTX2::*fpvR*_1-89_TAP	519 bp PCR fragment containing the *fpvR* promoter and 5′end of fpvR, joined to a C-terminal TAP-tag and cloned into Mini-CTX2	This study
pFLP2	pRO1600 ori, *sacB, flp* recombinase; Ap^R^/Cb^R^	[37]
pMP190::P_*fpvA*__*lacZ*	*fpvA* promoter cloned upstream of *lacZ* in pMP190; Cm^R^	[6]
pMP190::P_*pvdE*__*lacZ*	*pvdE* promoter cloned upstream of *lacZ* in pMP190; Cm^R^	[30,38]
pETDuet	Dual expression vector; Ap^R^	[39]
pETDuet::*fpvR*_1–89_	*fpvR*_1–89_ cloned into MCS2 of pETDuet	This study
pETDuet::*fpvI*	*fpvI* cloned into MCS1 of pETDuet	This study
pETDuet::*pvdS*	*pvdS* cloned into MCS1 of pETDuet	This study
pETDuet::*fpvI_fpvR*_*1–89*_	*fpvI* cloned into MCS1 and *fpvR*_1–89_ cloned into MCS2 of pETDuet	This study
PETDuet::*pvdS*_*fpvR*_*1–89*_	*pvdS* cloned into MCS1 and *fpvR*_1–89_ cloned into MCS2 of pETDuet	This study

### Co-purification of FpvR_1–89_ and FpvI or PvdS from *E. coli*

**Figure 3:**
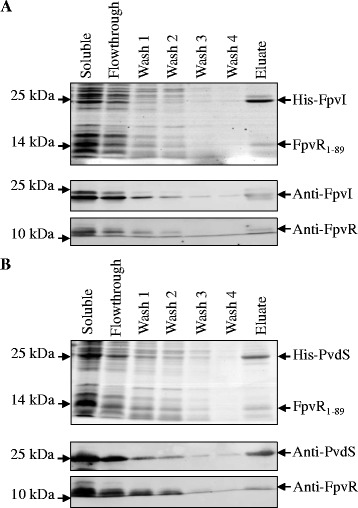
**Co-purification of FpvR**_**1–89**_ **with either His**_**6**_ **-FpvI or His**_**6**_ **-PvdS from** ***E. coli*** **.** Soluble protein was obtained from *E. coli* MC1061 (DE3) co-expressing either His_6_-FpvI and FpvR_1-89_ or His_6_-PvdS and FpvR_1–89_. Protein was purified by nickel affinity chromatography via the His_6_-tags on PvdS and FpvI and analyzed by SDS-PAGE (top panels) and Western blotting (lower panels) using anti-FpvI, anti-PvdS or anti-FpvR antibodies as shown. **(A)** Co-purification of FpvR_1–89_ with His_6_-FpvI; **(B)** co-purification of FpvR_1-89_ with His_6_-PvdS. The positions of molecular weight markers are shown.

The pETDuet co-expression system was used to investigate the interaction of FpvI and PvdS with FpvR_1–89_, via purification of hexahistidine-tagged FpvI or PvdS. FpvR_1–89_ co-purified with His_6_-FpvI and with His_6_-PvdS (Figure 3A and B). FpvR_1–89_ was not purified when His_6_-FpvI or His_6_-PvdS were absent (Additional file 1: Figure S2). In a reciprocal experiment, untagged FpvI and PvdS co-purified with His_6_-FpvR_1–67_ that contains only the 67 N-terminal residues of FpvR (Additional file 1: Figure S3). These findings demonstrated that the cytoplasmic region of FpvR forms stable complexes with either FpvI or PvdS when co-expressed in *E. coli*.

### Identification of mutations that increase sigma factor activity in the presence of FpvR_1-89_

The above data demonstrated binding and inhibition of FpvI and PvdS by FpvR_1–89_. To further investigate interactions of each sigma factor with FpvR_1–89_ we generated, selected and characterized mutations that increased the activity of FpvI and PvdS in the presence of FpvR_1-89_. Error-prone PCR was used to introduce random mutations into *fpvI* or *pvdS* and libraries of mutated genes were cloned into MCS1 of pETDuet co-expressing *fpvR*_*1–89*_. The mutagenesis method used minimizes bias in mutations [40] and sequence analysis of 9 independent clones (a total of 25 mutations) of mutagenized *pvdS* did not suggest any mutational bias (Additional file 1: Table S2). The resulting plasmid libraries were transformed into *E. coli* containing either P_*fpvA*_::*lacZ* or P_*pvdE*_::*lacZ*, to screen for gain-of-function mutations in *fpvI* and *pvdS* respectively. Mutants exhibiting increased activity in the presence of FpvR_1–89_ were identified on a qualitative basis as colonies showing increased β-galactosidase activity on agar plates supplemented with BCIG, as described in Methods.

**Figure 4:**
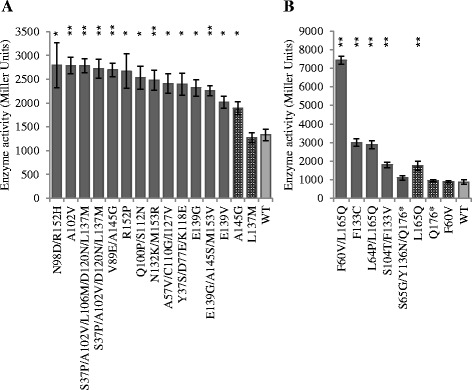
**The activity of FpvI and PvdS mutants in the presence of FpvR**_**1–89**_ **.** β-galactosidase assays were carried out with *E. coli* MC1061 (DE3) containing **(A)** pMP190::P_*fpvA*__*lacZ* or **(B)** pMP190::P_*pvdE*__*lacZ*, along with pETDuet expressing FpvR_1–89_ and **(A)** mutant FpvI or **(B)** mutant PvdS. Dark grey bars: FpvI and PvdS mutants generated by error prone PCR. Patterned bars: FpvI and PvdS mutants engineered by site directed mutagenesis. Light grey bars: WT FpvI or PvdS. Mutants that were further investigated in Figure 5 are indicated in bold. Data were obtained from three biological replicates and error bars are ±1 SD. Statistically significant difference to WT according to Student's T-test is indicated: *p <0.05, **p <0.01.

Approximately 6% of FpvI mutant colonies and 0.5% of PvdS mutant colonies had increased sigma factor activity in this screen. Mutant *fpvI* and *pvdS* genes were sequenced and their *lacZ* activities quantified. The properties of each mutant confirmed to have increased sigma factor activity are summarized in Additional file 1: Table S3 and S4. The FpvI mutants contained one to five amino acid changes with an average of two, and PvdS mutants contained one to three amino acid changes with an average of two. The activities of the FpvI and PvdS mutants were generally significantly higher than those of WT FpvI or PvdS (Figure 4; Additional file 1: Table S3 and S4). One exception was mutant PvdS S65G/Y136N/Q176* that had clearly increased activity relative to WT PvdS on screening medium but not in quantitative assays.

Many of the mutant genes contained multiple mutations but all of the mutant genes contained at least one mutation in or around conserved region four. Two single mutations were engineered into *fpvI* and three into *pvdS* by site-directed mutagenesis in order to investigate the effects of the individual mutations on interactions of the sigma factors with FpvR_1–89_. These mutations were chosen because the corresponding amino acid residues were altered in more than one mutant, they were located in region four, or they apparently enhanced the activity of a change in region four. Of these mutants, FpvI A145G and PvdS L165Q had higher activity than WT, although the activity of the latter was not as high as in F60V/L165Q or L64P/L165Q double mutants. PvdS F60V alone showed no difference in activity from WT indicating that F60V only had an effect on activity in the presence of L165Q.

### Activity of FpvI and PvdS mutants in the absence of FpvR_1–89_

**Figure 5:**
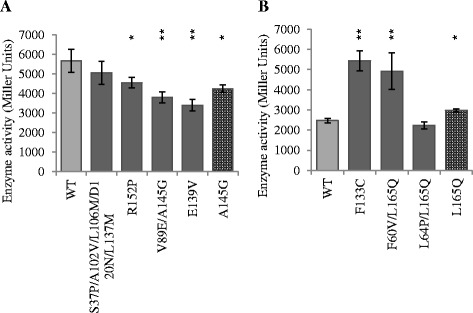
**The activity of FpvI and PvdS mutants in the absence of FpvR**_**1–89**_ **.** β-galactosidase assays were carried out with *E. coli* MC1061 (DE3) containing **(A)** pMP190::P_*fpvA*__*lacZ* or **(B)** pMP190::P_*pvdE*__*lacZ*, along with pETDuet expressing **(A)** mutant FpvI or **(B)** mutant PvdS. Dark grey bars: FpvI and PvdS mutants generated by error prone PCR. Patterned bars: FpvI and PvdS mutants engineered by site directed mutagenesis. Light grey bars: WT FpvI or PvdS. Data were obtained from three biological replicates and error bars are ±1 SD. Statistically significant difference to WT according to Student's T-test is indicated: *p <0.05, **p <0.01.

Increased activity of FpvI or PvdS mutants in the presence of FpvR_1–89_ could be due to reduced affinity of the mutant proteins for FpvR_1–89_ or to an intrinsic increase in sigma factor activity, which could arise from several factors including increased protein stability, improved affinity for core RNA polymerase, or stronger promoter DNA binding. To distinguish between reduced affinity for FpvR_1–89_ and intrinsically increased activity of the sigma factors, five FpvI and four PvdS mutants were compared with WT for activity in the absence of FpvR_1–89_. The activities of the FpvI mutants (Figure 5A) were not higher than WT FpvI; indeed, for four of these mutants the activity was lower than WT FpvI, which may indicate impaired function or destabilized protein folding as a result of the mutations. These data suggest that the increased activity of the FpvI mutants in the presence of FpvR_1–89_ (Figure 4A) is because in each case the mutations reduce the ability of FpvR_1–89_ to bind FpvI. In contrast, three of the PvdS mutants had significantly higher activity than WT PvdS in the absence of FpvR_1–89_ (Figure 5B). The increased activity of these mutants in the presence of FpvR_1–89_ (Figure 4B) may therefore be due at least in part to improved sigma factor function or protein stability.

### The mutations identified in FpvI cluster in region four

**Figure 6:**
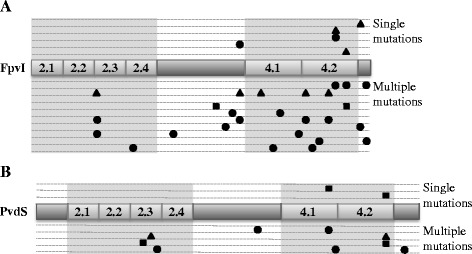
**The location of mutations in FpvI and PvdS that increased sigma factor activity in the presence of FpvR**_**1–89**_ **.** The approximate location of mutations are shown on the ECF sigma factor functional regions two (2.1-2.4) and four (4.1 and 4.2) of **(A)** FpvI and **(B)** PvdS according to the following classifications: ▲mutation(s) that gave higher activity in the presence, not absence of FpvR_1–89_; ■ mutation(s) that gave higher activity whether or not FpvR_1–89_ was present; ● mutation(s) that gave higher activity in the presence of FpvR_1–89_ and were not tested in the absence of FpvR_1–89_. Each mutant is represented on a different line.

The identified mutations were mapped onto the conserved regions of FpvI and PvdS [22]. The mutations clustered in region four of FpvI (Figure 6), including single mutations that increased the activity of FpvI in the presence of FpvR_1–89_, suggesting that this region is important for binding of FpvI by FpvR. No obvious clustering of mutations was observed for PvdS, however the sample size was small.

### Investigating the effects of FpvI and PvdS C-terminal deletions on activity

**Figure 7:**
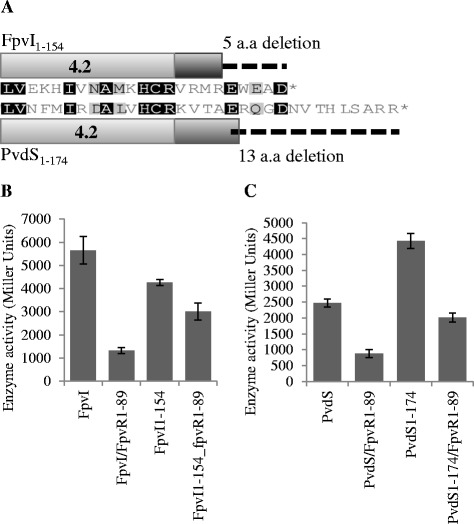
**The activity of FpvI and PvdS C-terminal deletion mutants. (A)** An alignment of FpvI showing the 5 amino acid C-terminal deletion and PvdS showing the 13 amino acid C-terminal deletion. **(B and C)** β-galactosidase assays were carried out with *E. coli* MC1061 (DE3) containing pMP190::P_*fpvA*__*lacZ* or pMP190::P_*pvdE*__*lacZ*, along with pETDuet expressing FpvR_1–89_ and C-terminal deletion mutants of **(B)** FpvI or **(C)** PvdS. Values are compared to WT FpvI and PvdS and were obtained from three biological replicates. Error bars are ±1 SD.

The truncation of PvdS by 12 amino acids due to the mutation Q176* had no effect on activity in quantitative assays (Figure 4B). To further investigate the role of the amino acids beyond region four in sigma factor activity, five amino acids were removed from the C-terminal end of FpvI (giving construct FpvI_1–154_) and 13 amino acids were removed from the C-terminal end of PvdS (PvdS_1–174_) (Figure 7A). FpvI_1–154_ had higher activity than WT FpvI in the presence of FpvR_1-89_, and lower activity than WT FpvI in the absence of FpvR_1–89_ (Figure 7B). This indicated that the amino acids beyond the C-terminal of region four are involved in binding to FpvR_1–89_. PvdS_1–174_ had higher activity than WT in both the presence and absence of FpvR_1–89_ (Figure 7C) suggesting that the removal of the C-terminal 13 amino acids improved protein stability or sigma factor function.

## Discussion

Interactions between ECF sigma and anti-sigma factor proteins have been experimentally demonstrated for a small number of systems [23-27,41]. FpvR is unusual amongst anti-sigma factors in that it inhibits the activities of two different sigma factors, FpvI and PvdS [6,10]. Genetic evidence has indicated that FpvR interacts directly with both FpvI and PvdS [28]. Using the TAP-tag approach we have now demonstrated that the cytoplasmic region of FpvR does indeed bind to PvdS in *P. aeruginosa*, with binding being stable enough to permit co-purification of PvdS with the FpvR_1–89_-TAP construct. A key aspect of the TAP-tag methodology is that proteins are expressed at physiological levels, avoiding any artefacts that may result from the use of overexpression constructs and providing confidence that FpvR naturally binds PvdS. Binding of FpvR_1–89_-TAP to PvdS in conjunction with extensive genetic evidence for FpvR/FpvI interactions ([6,7,28], this study) allow us to conclude that FpvR also binds to FpvI, inhibiting its activity. However, we were unable to detect co-purification of FpvI with FpvR_1–89_-TAP (data not shown). This may be because the amount of FpvI in *P. aeruginosa*, which has not been measured, is much lower than the amount of PvdS (500–700 molecules per cell [42,43]), or because FpvR_1–89_ has lower affinity for FpvI than for PvdS, or due to limitations of the polyclonal FpvI antibody.

Co-expression of FpvR_1–89_ with either PvdS or FpvI in *E. coli* enabled purification of FpvR_1-89_–sigma factor complexes, providing further evidence that the cytoplasmic region of FpvR can bind to both FpvI and PvdS. The use of reporter gene constructs showed that binding inhibited sigma factor activity. The pETDuet vector used for these experiments gives approximately equal expression of co-expressed genes (in this case *fpvR*_*1–89*_ with either *fpvI* or *pvdS*). The strong inhibition of sigma factor activity seen in reporter gene assays (Figure 2), as well as the approximately equi-molar amounts of FpvR_1–89_ and FpvI/PvdS obtained following co-purification (Figure 3), suggest that FpvR has a high affinity for each of its partner sigma factor proteins despite their very different sequences. Comparisons of the small number of ECF sigma factor structures available indicate that all have a similar structure involving two alpha helix bundles, corresponding to regions two and four, connected by a disordered region [23-26,41]. It is likely that FpvI and PvdS have the same overall structure with sigma factor-specific features that are recognized by FpvR.

A bacterial 2-hybrid system was used previously to identify mutations that interfere with FpvR-sigma factor binding [28] although mutagenesis was not carried out on PvdS and was only performed on the C-terminal section of FpvI (FpvI_95–159_). A disadvantage of this system is that loss of protein-protein interaction can occur through mutations that have a non-specific effect on protein structure or amount and indeed the majority of mutations identified in that study resulted in reduced amounts of the mutant protein. One amino acid change (L103P) was identified that reduced affinity of FpvI for FpvR in the bacterial 2-hybrid system, while not affecting the amount of FpvI protein. We reasoned that selection for gain-of-function mutations using full-length FpvI and PvdS would identify further residues involved in sigma-FpvR interactions, while excluding mutations that caused reduced amounts of sigma factor or disrupted the overall protein structure. We therefore used the pETDuet co-expression system, in conjunction with error-prone PCR and reporter gene assays, to identify gain-of-function mutations that increased the activity of full length FpvI and PvdS in the presence of FpvR_1–89_. A number of mutations (for example, FpvI E139V/G; FpvI N132K/M153R; PvdS F133C/V and PvdS L165Q) were obtained in independent screens, from different pools of mutagenized genes. This parallel evolution suggests that these mutations were particularly effective in increasing sigma factor activity in the presence of FpvR_1–89_. Additional mutant genes containing single mutations were engineered by site-directed mutagenesis. Single mutations in FpvI (residues A102V, E139V/G, R152P and A145G) increased the activity of FpvI in the presence of FpvR_1–89_, implicating these residues in FpvR – FpvI interactions. None of the mutant FpvI variants tested had higher activity than WT in the absence of FpvR_1–89_ (Figure 5) showing that most if not all of the FpvI mutations specifically affect interactions with FpvR.

Three of the four PvdS mutants tested had higher activity than WT PvdS in the absence as well as the presence of FpvR_1–89_. This suggests that the effect of these mutations was at least partially due to increased sigma factor function or protein stability. In the absence of FpvR, PvdS expressed in *E. coli* forms inclusion bodies [44] and it may be that these mutations improve the solubility of PvdS or alternatively its affinity for core RNA polymerase or promoter DNA. PvdS L64P/L165Q was the only PvdS mutant to show increased activity in the presence of FpvR_1–89_ and similar activity to WT PvdS in the absence of FpvR_1-89_, suggesting that this combination of mutations disrupted interactions with FpvR_1–89_.

Screening for increased activity of FpvI and PvdS in the presence of FpvR_1–89_ gave different outcomes for the two sigma factors. FpvI mutants were obtained at a high frequency (approximately 6% of colonies) and the mutants tested had similar or lower activity than WT FpvI in the absence of FpvR_1–89_. PvdS mutations were obtained at a lower frequency (approximately 0.5% of colonies) and three of the four tested had higher activity than WT PvdS in the absence of FpvR_1–89_. One explanation for these differences is that mutations which disrupt interactions of PvdS with FpvR_1–89_ also reduce the activity of PvdS and so would not be identified in our gain-of-function screen. Alternatively, interaction of FpvI with FpvR_1–89_ may involve a relatively small number of amino acid residue interactions and disrupting one of these significantly reduces the affinity of FpvI for FpvR, whereas PvdS-FpvR_1–89_ interactions may involve a larger number of weaker interactions and disrupting only one of these may not give a detectable reduction in inhibition by FpvR_1–89_.

All the FpvI mutants that showed enhanced activity in the presence of FpvR_1–89_ had mutations in or around region four (Figure 6). This provides clear evidence for the role of this region in FpvR binding, consistent with earlier findings [28]. The PvdS mutants that showed enhanced activity in the presence of FpvR also had mutations in region four. However, fewer mutants were identified and the majority of those tested had enhanced sigma factor function in the absence of FpvR_1–89_ making the role of region four in binding to FpvR less clear-cut. Region four in each of FpvI and PvdS is predicted to be involved in promoter recognition at the −35 site [20,22,45] and structural data for *E. coli* housekeeping sigma 70 shows all regions of sigma 70 to make contact with core RNA polymerase [45]. Therefore, FpvR binding to region four might sterically interfere with both promoter recognition and recruitment of core RNA polymerase. There is considerable variability amongst ECF sigma factors in the region bound by the cognate anti-sigma factor which can be both region two and four [23-25], region two alone [26] or region four alone [27]. FpvR binding and sequestering any of these regions would likely disrupt recruitment of core RNA polymerase.

Although region four of FpvI is clearly critical for interactions with FpvR_1–89_, our results also suggest some involvement of region two in either improving sigma factor function or in interactions with FpvR_1–89_. For example, changes at residue S37 in region 2.2 of FpvI arose in three separate screens in combination with additional mutations. The PvdS double mutants F60V/L165Q and L64P/L165Q showed markedly higher activity than the PvdS L165Q mutant in the presence of FpvR_1–89_. For PvdS F60V/L165Q the effect appeared to be synergistic because the individual mutants showed no difference in activity to WT PvdS. These results, together with the fact that no improved mutants were identified containing only a mutation in region two, suggest that changes in region two stabilized the protein or improved sigma factor function.

One mutation identified in our initial screen was PvdS Q176*, although this mutation did not increase activity of PvdS in quantitative assays. This suggested that the 12 amino acids at the C-terminus of PvdS, which occur after region four, are not required for sigma factor function. An engineered mutant where the last 13 amino acids of PvdS were deleted showed higher activity than WT while apparently retaining interaction with FpvR_1–89_. It is possible that the residues at the C-terminal end of region four act as a signal targeting PvdS for degradation [46]. Removal of these residues could improve stability of the protein. Alternatively it could enhance interactions with core RNA polymerase if the flexibility of these residues decreases the strength of interaction. Deletion of five residues at the C-terminal of FpvI resulted in increased sigma factor activity in the presence of FpvR_1–89_ suggesting that this region also contributes to binding by FpvR_1–89_.

## Conclusions

The cytoplasmic domain of FpvR forms stable interactions with PvdS in both *P. aeruginosa* and *E. coli*, and with FpvI in *E. coli*, despite the relatively low sequence identity of the sigma factors. Region four of FpvI as well as the C-terminus of this protein are of primary importance in binding to FpvR and we have identified four amino acid residues in FpvI that are likely to play a key role in its interaction with FpvR. It is likely that FpvI and PvdS share the same general tertiary structure that, in combination with specific residue interactions, is recognized by FpvR although we were unable to identify any single mutations that clearly reduced the affinity of PvdS for FpvR_1–89_. Our data are consistent with a model whereby FpvR inhibits FpvI and PvdS by occluding their binding to core RNA polymerase.

## Methods

### General methods

Bacterial strains used in this study are listed in Table 1. Bacteria were routinely grown in LB medium or on LB agar at 37°C. *P. aeruginosa* was grown using King’s B medium [47] for preparation of protein extracts. Media were supplemented with ampicillin (50 μg · mL^−1^), chloramphenicol (30 μg · mL^−1^), carbenicillin (300 μg · mL^−1^), tetracycline (25 μg · mL^−1^) or gentamicin (300 μg · mL^−1^) as required. *E. coli* MC1061 (DE3) was derived by lysogenizing *E. coli* MC1061 with λDE3 prophage (Novagen) using the manufacturer’s protocol.

### Genetic manipulations

DNA constructs were made using PCR, with the PCR primers (Additional file 1: Table S1) containing introduced restriction sites to enable cloning. A PCR fragment comprising the *fpvR* promoter and the 5′ end of *fpvR*, encoding the predicted cytoplasmic part of FpvR (residues 1–89; designated *fpvR*_*1–89*_) was amplified from *P. aeruginosa* PAO1 genomic DNA using primers miniPfpvRXbaIfor and fpvRNEcorRIrev. This fragment was ligated (via the introduced *Eco*RI site) to an *Eco*RI-digested fragment encoding a C-terminal TAP tag (C-TAP) that had been amplified with primers CTAPEcoRIfor and CTAPBamHIrev from plasmid pCTAPi [48]. The resulting *fpvR*_1–89_-TAP fragment was cloned into pUCP23 [35] and miniCTX2 [36] using the introduced *Xba*I and *Bam*HI restriction sites. Plasmids were introduced into *P. aeruginosa* by transformation or by conjugation from *E. coli* S17-1, and miniCTX2 vector sequences were then excised from the integrated miniCTX2 construct, as described previously [36]. pETDuet constructs were made by amplifying *fpvR*_1–89_, *pvdS* and *fpvI* from *P. aeruginosa* PAO1 genomic DNA using appropriate primers (Additional file 1: Table S1) and then cloning the resulting PCR fragments into pETDuet-1 [39]. The fidelity of all constructs was verified by DNA sequencing.

### Protein purification

Proteins were purified from *P. aeruginosa* using the tandem affinity tag (TAP tag) method [29], with all purification steps carried out at 4°C. Following 24 h incubation at 37°C, bacteria were collected from 1.2 L of culture by centrifugation (6160 × g, 15 min), resuspended in chilled lysis buffer (10 mM Tris-Cl [pH 8.0], 75 mM NaCl, 0.05% (v/v) Tween 20, 20% (v/v) glycerol, 25 mM KCl, 2 mM EDTA [pH 8.0], 0.5 mM DTT, 0.5 mM PMSF) (10 mL) and lysed by sonication (Sonics Vibra Cell). The lysed cells were centrifuged (16,000 × g, 30 min) and the supernatant was applied to an IgG sepharose column (Amersham Biosciences/GE Healthcare) (0.5 mL) that had been equilibrated with lysis buffer. The column was washed with approximately 20 volumes of lysis buffer then equilibrated with AcTEV cleavage buffer (Invitrogen). The column-bound protein was treated with 100 U of AcTEV protease (Invitrogen) (12 h). The released protein was collected, mixed with an equal volume (1 mL) of calmodulin binding buffer (10 mM Tris-Cl [pH 8.0], 75 mM NaCl, 2 mM CaCl_2_, 0.05% (v/v) Tween 20, 10 mM β-mercaptoethanol, 0.5 mM PMSF) containing Complete™ protease inhibitor Mini tablet (Roche) (1 tablet/100 ml of buffer). It was then applied to a calmodulin sepharose column (Amersham Biosciences/GE Healthcare), washed twice with calmodulin binding buffer and then washed using fresh calmodulin binding buffer in which the concentration of Tween 20 had been amended to 0.02% (w/v). Elution buffer (10 mM Tris-Cl [pH 8.0], 100 mM NH_4_HCO_3_, 10 mM EGTA, 10 mM β-mercaptoethanol) (1.2 mL applied in 6 aliquots) was then added and the protein collected.

Proteins expressed as hexahistidine fusions in pETDuet were purified from *E. coli* by nickel-affinity chromatography. Cultures grown for 16 h were used to inoculate fresh media to OD_600_ = 0.1. Expression was induced at OD_600_ = 0.6 using a final IPTG concentration of 14 μg · mL^−1^ at 18°C for 16 h. The cells were collected by centrifugation and then resuspended in binding buffer (50 mM sodium phosphate [pH 8.0], 300 mM NaCl). Cell lysis was carried out using sonication as described above. The soluble fractions were obtained by centrifugation and applied to nickel-affinity chromatography resin (BioRad) equilibrated in binding buffer. The resin was washed four times in wash buffer (50 mM sodium phosphate [pH 8.0], 300 mM NaCl, 30 mM imidazole) prior to elution in elution buffer (50 mM sodium phosphate [pH 8.0], 300 mM NaCl, 250 mM imidazole).

Proteins were analyzed by SDS-PAGE and Western blotting as described previously [14,43] using monoclonal antibodies against CBP (Upstate, Millipore), PvdS [49], the N-terminal (cytoplasmic) portion of FpvR [14], and a polyclonal antibody against FpvI (generated in-house), applied to the membrane sequentially.

### β-galactosidase reporter gene assays

Bacteria were inoculated in duplicate into 150 μL overnight cultures in LB amended with appropriate antibiotics and 0.4% glucose in wells of a 96-well flat bottom microtiter plate and grown for 16 h, 200 rpm at 37°C. Wells of a fresh microtiter plate, each containing 200 μL LB, antibiotics and 0.2% glucose, were inoculated with 15 μL of the overnight culture and incubated for 4 h, 200 rpm at 30°C. Portions (100 μL) of the resulting micro-cultures were inoculated into wells of a microtiter plate containing induction media (100 μL LB, antibiotics, 0.05 mM IPTG, 0.2% glucose) and incubated for 1 h, 200 rpm at 30°C. Absorbance was measured at OD_600_ using an Enspire plate reader (Perkin Elmer). For the assay, 20 μL of each induced culture were added to sodium phosphate buffer (84 μL, 40 mM, pH 7.0) containing ZOB buffer (46 μL) [50] and immediately incubated in the plate reader at 37°C without shaking. Absorbance readings at OD_420_ were taken at time zero and then every 2 minutes for 30 minutes. Absorbance readings for LB control wells were subtracted from the data. The enzyme activity for each well was calculated using the Miller equation [51]. Each assay was carried out in triplicate.

### Mutagenic PCR

A mutagenic PCR protocol was developed using Red Hot Taq polymerase (ABgene) that lacks 3′-5′ exonuclease activity, and error prone buffer conditions based on those described in [40]. The error prone PCR protocol used has been shown to minimize the intrinsic nucleotide substitution bias of Taq polymerase and the occurrence of mutation hotspots. Each reaction contained 2 μL 10 × mutagenic PCR buffer (70 mM MgCl_2_, 500 mM KCl, 100 mM Tris–HCl, [pH 8.2]), 2 μL 10 × dNTP (2 mM dGTP, 2 mM dATP, 15 mM dCTP, 10 mM dTTP), 1 μL 6 pmol · μL^−1^ forward primer, 1 μL 6 pmol · μL^−1^ reverse primer, 11 μL ddH_2_O, 2 μL 5 mM MnCl_2_, 0.4 μL Red Hot Taq polymerase, and 0.6 μL 5 ng · μL^−1^ template DNA. The reactions were incubated for 4 minutes at 94°C, followed by 30 cycles of 30 seconds at 94°C, 30 seconds at 55°C and 2 minutes at 72°C. For mutagenesis of *pvdS*, mutagenic PCR was carried out using primers pvdSBamHIfor and pvdSSalIrev (Additional file 1: Table S1), with pETDuet::*pvdS* as template. For mutagenesis of *fpvI*, mutagenic PCR was carried out using primers fpvIBamHIfor and fpvISacIstoprev (Additional file 1: Table S1) with pETDuet::*fpvI* as template. Libraries of mutated PCR products were cloned into pGEM-T Easy (Promega) and then sub-cloned into pETDuet_*fpvR*_1–89_ for screening. Three separate *pvdS* and *fpvI* libraries were made. Libraries were grown on LB agar containing 24 μg · mL^−1^ IPTG and 120 μg · mL^−1^ BCIG. Mutants that had enhanced reporter plasmid activity were identified as deeper blue colonies and were analyzed by DNA sequencing and β-galactosidase assay.

Overlap PCR was used to generate single mutations in *fpvI* and *pvdS* genes. The genes were amplified from PAO1 genomic DNA using Phusion High-Fidelity polymerase (Thermo Scientific) in two fragments using primers (Additional file 1: Table S1) that created a 25 bp overlap. Overlap PCR was performed using Biomix Red master mix (Bioline) with the two purified template fragments at equi-molar concentration totaling 100 ng in a volume of 40 μL. The reactions were initially incubated in the absence of primers for 2 minutes at 95°C, followed by 15 cycles of 30 seconds at 95°C, 30 seconds at 55°C, 30 seconds/kb at 72°C. The full length primers were then added in 20 μL of Biomix Red, adjusted for the final reaction volume of 60 μL. The reactions were incubated for an additional 15 cycles of 30 seconds at 95°C, 30 seconds at 67°C, 30 seconds/kb at 72°C, followed by 5 minutes at 72°C.

## Additional file

Additional file 1:
**Supplemental tables S1-S4 and figures S1-S3 associated with this manuscript.**

## Abbreviations

CBP: Calmodulin binding protein
ECF: Extracytoplasmic function
MCS: Multiple cloning site
TAP-tag: Tandem affinity purification tag
WT: Wild-type
