# Supplementary material for: Interactions between an anti-sigma protein and two sigma factors that regulate the pyoverdine signaling pathway in Pseudomonas aeruginosa
Source: BMC Microbiol. 2014 Nov 30;14:287. doi: 10.1186/s12866-014-0287-2 (PMC4256889; doi:10.1186/s12866-014-0287-2)
Supplement: Additional file 1: — Supplemental tables S1-S4 and figures S1-S3 associated with this manuscript. [file 12866_2014_287_MOESM1_ESM.pdf]

Table S1. Oligonucleotide primers used in this study

Name	Sequence
miniPfpvRXbaIfor	CCCTCTAGATACGCGTCGTGGGCGACATC
fpvRNEcorRIrev	AAAGAATTCGCTCTTCCAGCCCCTGCGGC
CTAPEcoRIfor	AAAGAATTCATGGAGAGCAGCAGATGGAA
CTAPBamHIrev	AAAGGATCCTGGCCCAGCTGGCCTCACTT
fpvIBamHIfor	GGGATCCATTGGAAAACCATTATCGGGAGC
fpvISacIstoprev	GGAGCTCTCAGTCGGCTTCCCATTCG
pvdSBamHIfor	CCACAGCCAGGATCCGATG
pvdSSalIrev	GGGGTCGACGCGGGCGCTGAGATGG
CfpvIHindIIIrev	CGGAAGCTTTCAGCGCATGCGCACCCGACA
CpvdSHindIIIrev	CGGAAGCTTTCACTCGGCGGTGACCTTGCG
FpvIintfor (C409A)	TTTCCACCATGCTGCGGGAAATATT
FpvIintrev (C409A)	TCCCGCAGCATGGTGGAAAAGC
FpvIintfor (C434G)	CGACAGTGCTTCATCCCGTTGACGA
FpvIintrev (C434G)	TCGTCAACGGGATGAAGCACTGT
PvdSintfor (T177G)	ACTTCGTCGGTCAAGGCACAGCTCA
PvdSintrev (T177G)	TGAGCTGTGCCTTGACCGACG
PvdSintfor (T494A)	ATGATCCGCGACGCCCAGGTGCACT
PvdSintrev (T494A)	GGCAGTGCACCTGGGCGTC
PvdSintfor (C526T)	AAGGTCACCGCCGAGCGCTAGGG
PvdSintrev (C526T)	CCCTAGCGCTCGGCGGTGA

Table S2. Spectrum of mutations obtained following mutagenic PCR.

Clone	Nucleotide Position	Basepair change
Clone 1	2722496	AT→AT
	2722624	delC
	2722728	GC→AT
Mutant 2	2722031	TA→AT
	2722368	GC→AT
	2722556	CG→AT
	2722211	CG→AT
	2722544	GC→TA
Mutant 3	2722139	CG→AT
	2722240	AT→TA
	2722624	CG→AT
	2722191	GC→TA
	2722288	TA→AT
	2722646	TA→AT
Mutant 4	2722111	GC→AT
	2722240	TA→AT
	2722455	AT→TA
	2722204	CG→AT
	2722316	TA→AT
	2722528	CG→AT
Mutant 5	2722419	delT
	2722461	AT→TA
Mutant 6	2722054	AT→TA
	2722644	AT→TA
Mutant 7	2722094	AT→GC
	2722284	AT→GC
	2722419	TA→CG
	2722128	GC→TA
	2722346	AT→TA
	2722449	TA→AT
	2722211	GC→AT
	2722377	AT→TA
Mutant 8	2722522	TA→AT
	2722541	AT→GC
Mutant 9	2722353	TA→AT
	2722367	AT→GC
	2722388	CG→TA
	2722455	AT→GC
	2722631	TA→CG
	2722688	AT→GC

Mutant 10	2722486	GC➔AT
	2722348	GC➔AT
Mutant 11	2722391	GC➔AT
Mutant 12	2722373	CG➔TA
Mutant 13	2722457	GC➔CG

Mutagenic PCR of the *pvdS* gene was carried out, PCR products cloned into pGEM-TEasy, and the sequences of individual clones (1 to 13) determined as described in the main text. Nucleotide position, position in the *P. aeruginosa* genome where the sequence of the mutated gene differs from the reference sequence (v2.pseudomonas.com); base-pair change, the change from wild-type to mutant sequence; del, base-pair deletion.

Table S3. FpvI mutants showing increased activity in the presence of FpvR$_{1-89}$

	Nucleotide change	Amino acid change	Region	Repeated mutation [a]	Activity + FpvR$_{1-89}$ (SE)	Activity – FpvR$_{1-89}$ (SE)
	None				1330 (126)	5655 (598)
FpvI	C170T	A57V	2.4		2409 (207)	ND[b]
	T328G	C110G	4.1			
	A379G	I127V	4.2			
	G455C	R152P	after 4.2	*	2675 (361)	4550 (262)
	T396A	N132K	4.2		2477 (211)	ND
	T458G	M153R	after 4.2	*		
	C110A	S37Y	2.3	*	2396 (236)	ND
	C231A	D77E	before 4.1			
	A352G	K118E	4.1			
	A292G	N98D	before 4.1		2794 (469)	ND
	G455A	R152H	after 4.2	*		
	A416T	E139V	4.2	*	2017 (128)	3393 (303)
	A416G	E139G	4.2	✓	2318 (176)	ND
	T109C	S37P	2.3	✓	2725 (198)	ND
	C305T	A102V	before 4.1	✓		
	G358A	D120N	4.2	✓		
	C409A	L137M	4.2	✓		
	A299C	Q100P	before 4.1		2531 (243)	ND
	G335A	S112N	4.1			
	C305T	A102V	before 4.1	✓	2787 (174)	
	T266A	V89E	before 4.1		2696 (144)	3793 (276)
	C434G	A145G	4.2	*		
	T109C	S37P	2.3	✓	2780 (148)	5052 (596)
	C305T	A102V	before 4.1	✓		
	T316A	L106M	4.1			
	G358A	D120N	4.2	✓		
	C409A	L137M	4.2	✓		
	A416G	E139G	4.2	✓	2257 (101)	ND
	G433T	A145S	4.2	*		
	A457G	M153V	after 4.2	*		
	C409A	L137M	4.2	SDM	1274 (105)	ND
	C434G	A145G	4.2	SDM	1890 (135)	4238 (186)

[a] The same amino acid change was identified in more than one mutant (✓) or the same amino acid residue was changed in more than one mutant (*) or the mutation was introduced by site directed mutagenesis (SDM). Mutants are separated by a line and mutants identified in different libraries are separated by a thickened line.
[b] ND = not determined.

Table S4. PvdS mutants with increased activity in the presence of $FpvR_{1\text{-}89}$

	Nucleotide change	Amino acid change	Region	Repeated mutation [a]	Activity + $FpvR_{1\text{-}89}$ (SE)	Activity – $FpvR_{1\text{-}89}$ (SE)
	None				876 (124)	1144 (53)
PvdS	A193G	S65G	2.3		1104 (119)	ND[b]
	T406A	Y136N	4.1			
	C526T	Q176*(stop)	after 4.2			
	T178G	F60V	2.3		7423 (221)	4922 (905)
	T494A	L165Q	4.2	✓		
	T398G	F133C	4.1	*	3006 (209)	5440 (496)
	T191C	L64P	2.3		2875 (214)	2228 (175)
	T494A	L165Q	4.2	✓		
	T311A	S104T	3.2		1807 (149)	ND
	T397G	F133V	4.1	*		
	T178G	F60V	2.3	SDM	902 (59)	ND
	T494A	L165Q	4.2	SDM	1755 (244)	2972 (73)
	C526T	Q176*(stop)	after 4.2	SDM	948 (54)	ND

[a] The same amino acid change was identified in more than one mutant (✓) or the same amino acid residue was changed in more than one mutant (*) or the mutation was introduced by site directed mutagenesis (SDM). Mutants are separated by a line and mutants identified in different libraries are separated by a thickened line.
[b] ND = not determined.

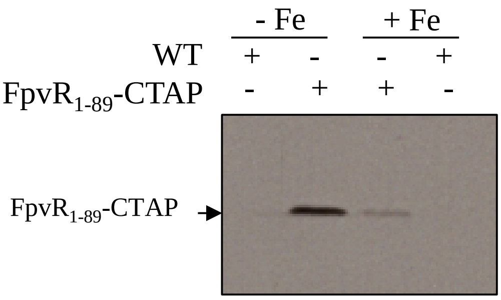

Figure S1. Iron regulation of FpvR$_{1\text{-}89}$–TAP in *P. aeruginosa*. Samples of *P. aeruginosa* PAO1 (WT) and PAO1 with chromosomally integrated *fpvR$_{1\text{-}89}$*–TAP grown in the presence and absence of 300 µM $FeCl_3$ were analysed by Western blotting with anti-CTAP. The position of 30 kDa FpvR$_{1\text{-}89}$-CTAP is shown.

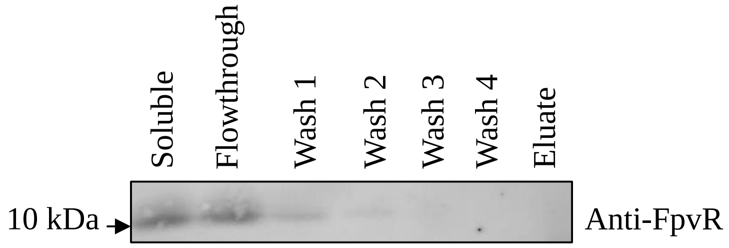

Figure S2. Mock purification of untagged $FpvR_{1\text{-}89}$. Soluble protein was obtained from *E. coli* MC1061 (DE3) carrying non-histidine tagged $FpvR_{1\text{-}89}$. The protein extract was subjected to a mock nickel affinity chromatography purification and analysed by Western blotting with anti-FpvR. The position of the 10 kDa molecular weight marker is shown.

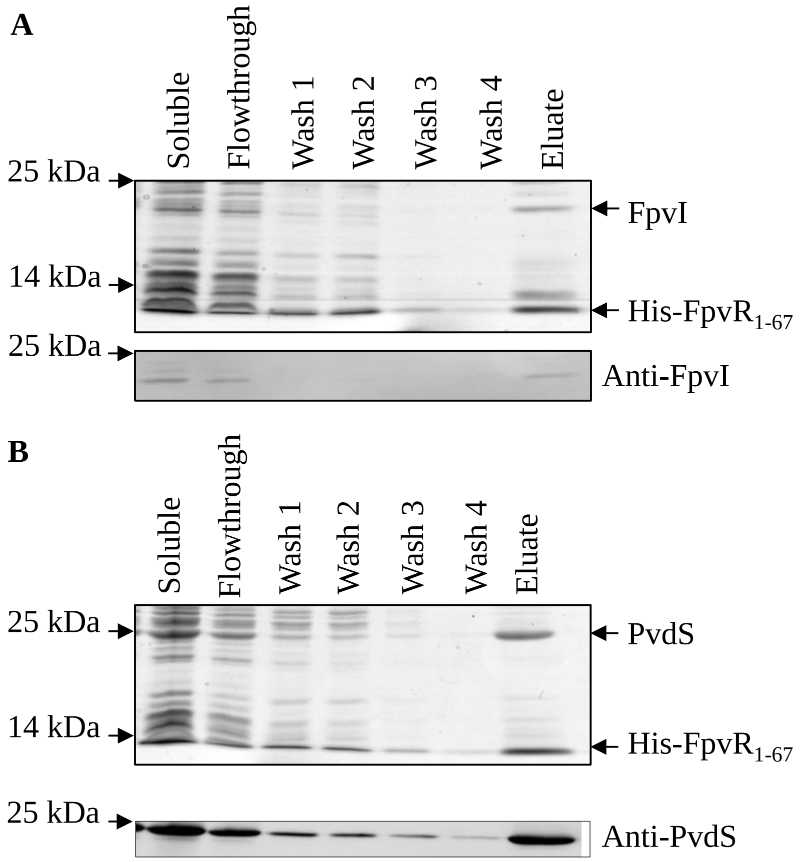

Figure S3. Co-purification of His_6-$FpvR_{1-67}$ with FpvI or PvdS from *E. coli*. Soluble protein was obtained from *E. coli* MC1061 (DE3) co-expressing His_6-$FpvR_{1-67}$ and FpvI or His_6-$FpvR_{1-67}$ and PvdS. Protein was purified by nickel affinity chromatography via the His_6-tag on $FpvR_{1-67}$ and analysed by SDS-PAGE and Western blotting with anti-FpvI, anti-PvdS. (A) Co-purification of His_6-$FpvR_{1-67}$ and FpvI; (B) co-purification of His_6-$FpvR_{1-67}$ and PvdS. The positions of the molecular weight markers are shown.
